# Ginger and Its Derivatives as Promising Alternatives to Antibiotics in Poultry Feed

**DOI:** 10.3390/ani10030452

**Published:** 2020-03-09

**Authors:** Mohamed E. Abd El-Hack, Mahmoud Alagawany, Hazem Shaheen, Dalia Samak, Sarah I. Othman, Ahmed A. Allam, Ayman E. Taha, Asmaa F. Khafaga, Muhammad Arif, Ali Osman, Ahmed I. El Sheikh, Shaaban S. Elnesr, Mahmoud Sitohy

**Affiliations:** 1 Department of Poultry, Faculty of Agriculture, Zagazig University, Zagazig 44511, Egypt; 2 Department of Pharmacology, Faculty of Veterinary Medicine, Damanhour University, Damanhour 22511, Egypt; dr_hazemshaheen3010@yahoo.com; 3 Department of Veterinary Forensic Medicine and Toxicology, Faculty of Veterinary Medicine, Damanhour University, Damanhour 22511, Egypt; dalia_samak@vetmed.dmu.edu.eg; 4 Biology Department, Faculty of Science, Princess Nourah bint Abdulrahman University, Riyadh 11671, Saudi Arabia; sialothman@pnu.edu.sa; 5 Department of Zoology, Faculty of Science, Beni-suef University, Beni-suef 65211, Egypt; allam1081981@yahoo.com; 6 Department of Animal Husbandry and Animal Wealth Development, Faculty of Veterinary Medicine, Alexandria University, Edfina 22578, Egypt; Ayman.Taha@alexu.edu.eg; 7 Department of Pathology, Faculty of Veterinary Medicine, Alexandria University, Edfina 22758, Egypt; Asmaa.Khafaga@alexu.edu.eg; 8 Department of Animal Sciences, College of Agriculture, University of Sargodha, Punjab 40100, Pakistan; arif.inayat@uos.edu.pk; 9 Department of Biochemistry, Faculty of Agriculture, Zagazig University, Zagazig 44511, Egypt; ali_khalil2006@yahoo.com (A.O.); mzsitohy@hotmail.com (M.S.); 10 Department of Public Health, Faculty of Veterinary Medicine, King Faisal University, Hofuf 31982, Saudi Arabia; aelsheikh@kfu.edu.sa; 11 Department of Poultry Production, Faculty of Agriculture, Fayoum University, Fayoum 63514, Egypt; ssn00@fayoum.edu.eg

**Keywords:** Ginger, microbial effect, chemical composition, body weight, carcass weight, birds

## Abstract

**Simple Summary:**

The present review updates the current knowledge about the beneficial effects of ginger and its derivatives as feed supplements to poultry feed, particularly its positive effects on the body weight, carcass traits, egg production and quality, reproductive performance, blood parameters, egg and meat quality and microbiological aspects as well as the economic efficiency.

**Abstract:**

Poultry enterprises have sustained rapid development through the last three decennaries. For which reason, higher utilization of antibacterial, either as therapeutic or growth promoting agents, has been accepted. Owing to the concern of developing bacterial resistance among populations towards antibiotic generations, accumulation of antibacterial remaining’s in chicken products and elevating shopper request for outcomes without antibacterial remaining’s, looking for unconventional solutions that could exchange antibacterial without influencing productiveness or product characters. Using natural alternatives including ginger, garlic prebiotics, organic acids, plant extracts, etheric oils and immune stimulants have been applied to advance the performance, hold poultry productiveness, prevent and control the enteric pathogens and minimize the antibacterial utilization in the poultry production in recent years. The use of a single replacement or ideal assemblage of different choices besides good supervision and livestock welfare may play a basic role in maximizing benefits and preserving poultry productiveness. The object of this review was to support an outline of the recent knowledge on the use of the natural replacements (ginger and its derivatives) in poultry feed as feed additives and their effects on poultry performance, egg and meat quality, health as well as the economic efficiency.

## 1. Introduction

On the present day, the non-controlled utilization of antibacterial agents in poultry feed is exposed to critical problems. There are critical factors that prohibit the usage of antibacterial agents such as the drug remaining in chicken meat as well as the drug resistance among bacterial populations. These outcomes have seriously controlled their usage in different nations owing to involvements associated with the spread of antibacterial-tolerant human pathogens and judicial achievement to control their usage in apparent many different nations [1,2,3,4,5]. A perfect prohibiting move on antibiotic usage in poultry feed was conducted into action on the 1st of January 2006 by way of the European Uniting Community; so, entirely of the antibacterial agents prescribed in sub-treating levels for growing promotion purposes (as antibacterial growing promotions, AGP’s) were detached [6]. For defeating the unfavorable productiveness and the expand susceptibleness to infections that originate from the withdrawal of antibacterial agents from chicken’ feeds, trials have been carried out to look for other practical alternatives. Feed supplements could be either nutritive or non-nutritive compounds that modulate the nutritive material accessibility in the feed finally lowering the expense of feed. As a substitute for the synthetic growth promoters, standard originated growing promoters like probiotics, prebiotics, botanical substances, and enzymes can be utilized to feed additives of the broiler chicken [7,8,9]. Usage of growth promoting agents of valid sources such as yeast cultivations, prebiotics, probiotics, enzymes, organic acids, botanically involved essential oils and extracts of some spices and herbs consequently appeared of interest in modern years [10,11,12,13,14,15,16]. Ginger rhizome *(Zingiber officinale)* was utilized as a delicacy spice or remedy. The usage of ginger as an alternative for antibacterial growth promoting agent is advantageous for the greater productiveness of poultry, improved appetite and palatability of feed, nutrient absorption, and advanced of gastric enzymes flow [17]. Either antibiotics or synthetic components totally utilized as feed add-on in livestock feed are under discussion since their probable toxicological impacts. In addition, there is an advancing direction of natural originated products for the purpose of reducing the usage of synthetic compounds [18]. So, medicinal herbs or their derivatives are presently believed intact and provide an achievable choice to fulfill customer needs and prevalent market contests [19,20]. 

*Zingiber officinale* Roscoe, relating to the Zingiberaceae family, commonly recognized as ginger, is a monocotyledonous herbaceous herb and one of the topmost popular feed-flavoring additive managed worldwide [21] Lately, various pharmacological characters of ginger, such as analgesic, anti-inflammatory, gastrointestinal modulating agent, antimicrobial and antioxidant characters have been mentioned [22]. Ginger could be a possible choice for popular synthetic growth promoting agents like antibacterials [23]. The featured ginger sense originated from the inconstant substances that composed of gingerols, shogaols and zingrone [24]. This review article aimed to shedlight the impacts of ginger or its derivatives as natural alternatives on growth performance, carcass characters, egg production and quality, reproductive performance, nutrient digestibility, immune response, some blood biochemical values, egg and meat quality and microbiological aspects of birds.

## 2. The Effect of Ginger Oil 

Ginger oil was originated from *Z. officinale* roots, which its components influenced according to the geographical area, roots condition and extraction protocols (freshness or dryness). The antifungal, antibacterial, anti-inflammatory, analgesic and immunomodulatory impacts of ginger oil have been distinguished in preclinical trials [25]. The harmlessness concerns of ginger oils are well-mentioned and are usually considered of being safe. By reason of broad pharmacological impacts of ginger oil, considerations to such element of valid combinations in the control of respiratory and gastrointestinal affections are of great importance.

## 3. Chemical Composition of Ginger Extract

Chemical components of ginger oil beside its potential anti-inflammatory and antioxidant were documented [26]. The major elements as discovered via Gas Chromatography–Mass Spectrometry (GC–MS) method were zingiberene which represented as 31% of the total area, curcumene (15.4%) and then sesquiphellandrene (14.02%). Ginger oil collected superoxide, hydroxyl and α, α-diphenyl-β-picrylhydrazyl (DPPH) radicals so, minimized tissue in vitro fat peroxidation. Ginger oil intraperitoneal inoculation caused inhibition of phorbol12-myristate-13-acetate initiated superoxide chemicals released by macrophages. While, oral application of ginger oil for at least 30 days, critically improved glutathione reductase, glutathione and superoxide dismutase (SOD) values [26]. Ginger oil’s product is altered from 1.0 to 3%, depending on the source of rhizomes [27]. Additionally, to the obtained essential oil’s, the chemical composition of ginger oils are influenced by the origin of rhizome, freshness or dehydration and extraction procedures.

**Figure 1:**
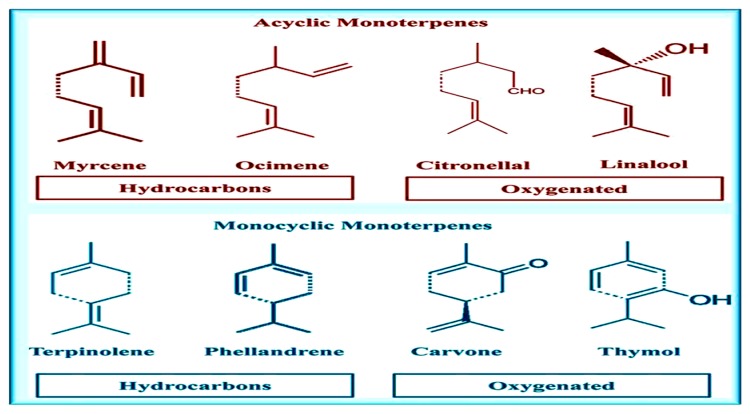
Structure of some monoterpenes.

**Figure 2:**
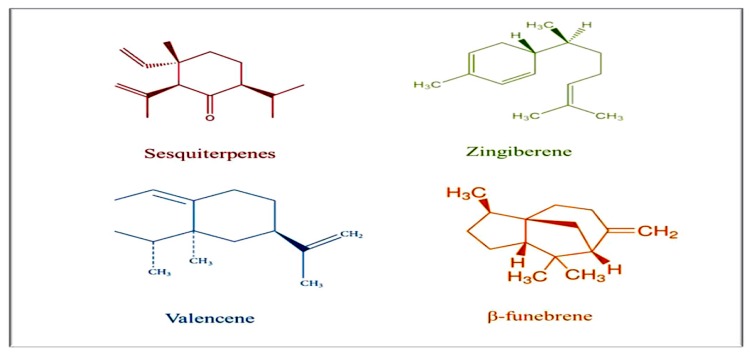
The major sesquiterpene components.

Singh et al. [28] extracted basic oil from *Zingiber officinale* fresh roots and described GC–MS protocol of the basic oil. They mentioned the presence of 69 ingredients, calculating for 96.93% of the whole oil. The vital ingredient was zingiberene (28.62%) then camphene (9.32%), then curcumene (9.09%) and finally β-phellandrene (7.97%). Analysis of the oleoresin mentioned the existence of 34 ingredients, measuring for 88.63% of the whole oleoresin. The critical ingredients were trans-6-shogaol (26.23%), trans-10-shogaol (13.0%), α -zingiberene (9.66%) and 10-gingerdione (6.80%). Furthermore, the basic oil was discovered to be 100% *Fusarium oxysporum* antifungal effect, while at the same time, oleoresin showed 100% antifungal in contact with *Aspergillus niger*. The foregoing showed advanced antioxidant action in sunflower oil in comparison with the basic oil or synthetic antioxidants (BHT and BHA). In addition, Sharma et al. [29] analyzed basic oil extracted from the raw roots of *Z. officinale* collected from Ghaziabad area in order to assess its antimicrobial action. The fresh roots were exposed to hydrodistillation to obtain the basic oil content which was analyzed by either GC or GC-MS protocols. The extracted oil was assessed for antimicrobial action by disc diffusion technique. Data showed that the extracted basic oil was distinguished by a high proportion of sesquiterpenes (66.66%) then monoterpenes (17.28%) and aliphatic ingredients (13.58%) as presented in Figure 1. The major sesquiterpene components (Figure 2) were zingiberene (46.71%) then valencene (7.61%), β-funebrene (3.09%) and selina-4(14),7(11)-diene (1.03%). Additionally, monoterpenes were distinguished as citronellyl n-butyrate (19.34%) then β-phellandrene (3.70%), camphene (2.59%) and α-pinene (1.09%). Also, Sasidharan et al. [30] discovered that inconstant oils came from two topmost prevailing cultivars from Sikkim namely, Bhaisa and Majulay, were detached, and distinguished by GC or GC-MS analytical protocols. Sixty ingredients representing about 94.9% and 92.6% of the Bhaisa and Majulay oils were recognized. The significant ingredients of Bhaisa oil were geranyl acetate (18.8%) then zingiberene (16.3%) and finally geranial (8.2%) and those of Majulay oil were zingiberene (19.8%) then geranial (16.5%). Compared to various ginger cultivar oils, the Bhaisa oil possessed senior constituents of oxygenated ingredients (43.1%).

Bhattarai et al. [31] aimed to establish the chemical constituents of necessary oil and biological assays from the crude ethanolic extract of ginger from Sindhupalchhowk, Tanahu and Gorkha region of Nepal. Ginger essential oils were extracted using hydro distillation, and having the yield percent; Gorkha: 1.9, Sindhupalchowk: 1.8 and Tanahu: 1.1. GC-MS technique showed the existence of 105, 85 and 88 ingredients from Sindhupalchowk, Tanahu and Gorkha ginger respectively, with the major constituntes, monoterpenes and sesquiterpenes derivatives. The Soxhlet extraction was performed for the 95 % ethanolic extraction from fresh ginger (EE) and the residue ginger after essential oil extraction.

Stoyanova et al. [32] studied the chemical ingredients of basic oils extracted either by steam distillation or water of ginger collected from Vietnam. The obtained basic oils were 2.05% and 2.1%, respectfully. The critical ingredients of the basic oil extracted by aqueous distillation protocol were curcumene (11.7%) then β-bisabolene (4.1%), and of the basic oil extracted by vapour distillation technique were curcumene (12.6 %) then α-zingiberene (10.3%), β-bisabolene (8.1%) and β-sesquiphellandrene (7.4%). 

**Figure 3:**
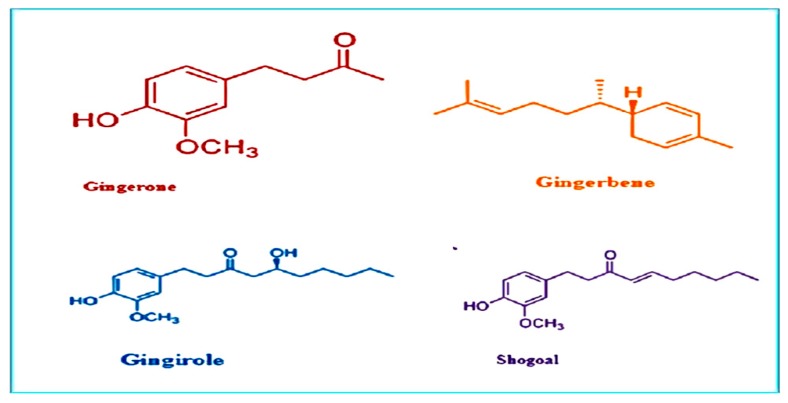
Chemical structure of important phytochemicals present in ginger.

El-Baroty et al. [33] demonstrated that basic oils extracted from the roots of *Zingiber officinale* were distinguished by TLC or GC–MS analytical protocols, and both their antioxidant and antimicrobial ingredients were mentioned by TLC supported bioautography techniques. Ginger oil was differentiated by great constituent of sesquiterpene hydrocarbons, including β-sesquiphellandrene (27.16%) then caryophyllene (15.29%), zingiberene (13.97%), α-farnesene (10.52%) and curcumene (6.62%). Chemical structure of important phytochemicals present in ginger is presented in Figure 3.

## 4. Beneficial Application of Ginger and Its Derivatives in Poultry Nutrition

**Figure 4:**
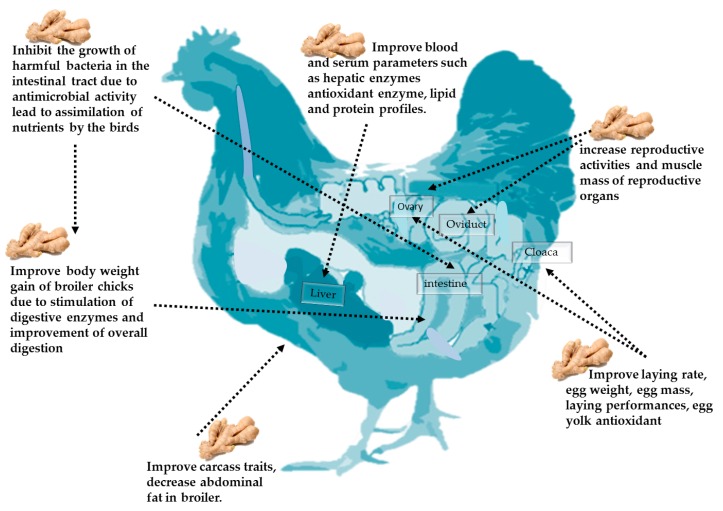
The beneficial application of ginger and its derivatives in poultry nutrition.

Beneficial effects of ginger and its derivatives in poultry nutrition are illustrated in Table 1 and Figure 4.

### 4.1. Effects of Ginger on Body Weight

Dieumou et al. [34] found that the study extended for at least seven weeks without variations in feed conversion ratio (FCR) and body weight (BW) profits among the birds feed (control) or 10 mg/kg/day (ginger oil). Shanoon et al. [35] performed a study to assess the impact of ginger oil on the microbial population and growth performance of broilers. Two hundred of both females and males, one-day-old chicks of Ross 308 were allocated in a perfect incidental form trial, into four treatments grouping for seven weeks; doses 0 (Control), 10 mg/kg/day, 20 mg/kg/day and 40 mg/kg/day. This study showed no clear variations in feed intake, FCR and BWG among the treated birds. Herve et al. [36] evaluated four different diets that contained (100 µl/kg BW of clean H_2_O as control, 50, 100, and 150 µl/kg BW of ginger basic oil sequentially). This research was conducted to assess the outcomes of ginger (*Zingiber officinale*) basic oil on growth parameters and biochemical oxidative/antioxidant features. Also, the histological configuration of testes and subsequently fertility features in Japanese quail. The main data mentioned that growth features were not clearly changed by essential oil regardless of dosage. The weight of left testis clearly elevated in basic oil treatments (100 or 150 µl/kg BW) when compared with the other groups.

Herve et al. [37] discovered the impacts of ginger (*Zingiber officinale*) basic oil on laying and growth features, beside cholesterol level and egg yolk antioxidant in Japanese quail. Eighty birds were randomly and equally allocated into four experimental groups treating orally and daily, 100 µl/kg BW clean H_2_O and 50, 100, and 150 µl/kg BW of ginger roots extracted basic oil. Outcomes exposed that FCR and body weight gain (BWG), were not clearly mentioned by oral administration of ginger rhizomes extracted basic oil. The oral application of ginger rhizomes extracted basic oil had no obvious outcomes on gizzard, heart, intestine and liver weights when put in comparison with the control data. Weight of eggs clearly improved in Japanese quails orally administered with ginger roots extracted basic oil no matter what the dose level with special attention to the control data. 

Habibi et al. [38] evaluated the outcomes of ginger addition on growth, antioxidant and carcass features as well as blood profile in broiler chicks exposed to heat stress environment (32 ± 2ºC for 8 hour each day). Male broiler chicks were equally and haphazardly allocated into 6 dietary treated groups. A basic diet with no add-on, basic diet coupled with vitamin E (100 mg/kg), basic diet plus either 7.5 or 15 g/kg of ginger root powder, and feeds enclosing 75 or 150 mg/kg BW of ginger basic oil. There were no clear changes among the treated groups concerning BW profits, feed intake features and FCR after 42 and 49 days of age. 

Mohamed [39] evaluated the growth parameters outcomes of broiler chicks nourished on graduated levels of garlic and ginger essential oils mixture during 42 days. Unsexed Arbor Acres (84 birds, 7days-old) were haphazardly and equally allocated into four experimental categories. The first group was fed a basic diet without feed add-on, the other treated groups were fed basic feeds mixed with different combinations of garlic and ginger essential oils at levels of (100 g ginger+100 g garlic)/ton, (200 g ginger + 200 g garlic)/ton and (300 g ginger + 300 g garlic)/ton. The data revealed that there were no clear variations of BWG and FCR among all treated groups.

Abd El-khalek and ELnaggar, [40] investigated the impact of ginger oil (*Zingiber officinale* Roscoe), black seed oil (*Nigella sativa* L.), thyme oil (*Thymus vulgaris* L.) and oregano oil (*Origanum vulgare* L.) as medicinal plants on the productive performance, blood constituents and DNA concentration of Gimmizah chicks. A total of 405 unsexed Gimmizah chicks (4 weeks of age) were randomly assigned to nine treatment categories of three simulates each. All chicks were raised in battery brooder placed in a temperature-controlled room until 16 weeks of age. Chicks in treatments 1, 2, 3 and 4 were fed a basic diet combined with 1 ml of ginger, black seed, thyme or oregano/kg diet, respectively. Chicks of treatments 5, 6, 7 and 8 were fed a basal diet with the addition of 0.5, 1.0, 1.5 and 2.0 ml/kg diet of an equal mixture of the previous medicinal plants together, respectively. Treatment 9 was used as a control. Results indicated that, the highest BW attain were recorded for the birds fed a basal diet with the addition of 1 ml oregano/kg diet. Also, Ghasemi and Taherpour [41] distinguished the outcomes of the basal diet including ginger essential oil (50, 100 or 200 mg/kg diet) plus mannan-oligosaccharide (2 g/kg diet) on growth features. One-day-old male broiler chicks (375 chicks) were haphazardly and equally assigned into five categories. Data revealed that the birds nourished on mannan-oligosaccharide plus ginger essential oil (200 mg/kg diet) mixture showed an improved BWG from the first to 42 days of age when compared with control data.

Tekeli et al. [42] utilized 105, one-day-old male broiler chicks (Ross 308) to compare different diets as growth promoter agents for 42 days. The chicks were randomly and equally allocated into seven dietary treatment groups (basic diet), (basic diet + 10 mg flavomycin/kg diet), (basic diet + 120 mg *Yucca schidigera* extract/kg diet), (basic diet + 120 mg *Oreganum vulgare* essential oil/kg diet), (basic diet + 120 mg *Thymus vulgaris* basic oil/kg diet), (basic diet + 120 mg *Syzygium aromaticum* basic oil/kg diet) and (basic diet + 120 mg *Zingiber officinale* essential oil/kg diet). Data revealed that *Z. officinale* advanced the BWG in comparison with other treated groups.

### 4.2. Effect of Ginger and Its Derivatives on Carcass Traits

Tekeli et al. [42] mentioned that the dietary supplement of herb extracts, (*Z. officinale*) stimulated growth feature and the population of beneficial microorganisms. Dietary supplement of *O. vulgare* or *Z. officinale* or *S. aromaticum* lowered the total measure of the digestive tracts of treated chickens, but advanced mass of jejunum. Also, it could be concluded that *Z. officinale* could replace antibacterials which have been prohibited to be used as growth promoting in animal nutrition. Herve et al. [36] confirmed that the liver weight was decreased in ginger basic oil-treated quails. 

Dieumou et al. [34] revealed that the majority of organ mass and carcass features were not suffered during the treatments, but for a drop in corresponding to the liver mass of chickens on garlic oil exposure when put in comparison with those data were given by either control or ginger oil. On the same way, a drop in the ratio of the head mass of chickens were administered basic oils was noticed to the control data. Dosages could cause a drop in relative mass of organs except for the gizzard and head when put in comparison to the control data. Male broilers were described less advanced than the females.

The oral application of ginger roots extracted basic oil (0, 0.25%, 0.50% and 0.75%) had insignificant outcomes on gizzard, heart, liver, and intestine considerable mass when differentiated to the control data, but the abdominal fat mass was significantly dropped in all exposed quails [43]. Mohamed [39] mentioned that carcass dressing and dependent meat quality features were not affected by adding of garlic and ginger essential oils mixture in the broiler diet.

### 4.3. Effect of Ginger and Its Derivatives on Egg Production and Quality

Osman [43] studied the effect of adding ginger oil in the diet on egg production and egg quality of Japanese quail in all periods of the experiment. Birds in the 8 weeks of age were distributed randomly on the four treatments, including four levels of ginger oil (0, 0.25%, 0.50% and 0.75%). The egg production and the production cumulative egg rate did not record also significant differences in the first period, but in the subsequent results of the experiment mentioned that there was no clear variation in the rate of treatments between three periods observed there significant superiority has added its oil treatments ginger in comparison with control as significant superiority in egg mass appeared to all treatments during the four periods of the experiment. So appeared obvious change in albumin weight when the fourth period, despite not appear significant differences in the first three periods and high significant in shell weight in the second period, while no significant differences did not appear in other periods for this trait, as well as did not record a significant differences in high yolk, yolk diameter, weight yolk, yolk index, high albumin and shell thickness.in addition to this study concludes that all additions ginger oil in Japanese quail diet led to significant superiority in egg production, no significant differences in quality characteristics of the eggs. Egg mass clearly advanced in Japanese quails exposed to ginger roots extracted basic oil without concerning the level of the dose with special attention to the control data [43].

Ginger roots extracted basic oil could be utilized in poultry to minimize lipid peroxidation status in germinal tissues and so, advanced the fertility status. In addition, oral application of 100–150 µl/kg BW of ginger roots extracted basic oil on laying Japanese quails, the highest outcomes on egg mass and lowered egg levels without any harmful factors on feed uptake and BW profits [36]. On the other hand, Herve et al. [37] noticed that egg productiveness features, and weekly weight of eggs were not clearly affected by oral application of ginger roots extracted basic oil. An et al. [44] investigated ginger extract on the productiveness, antioxidant and immunity features of laying hens. The control birds were fed the basic diet; the experimental birds were fedbasic diets plus 0.1% ginger extract. Ginger extract clearly advanced laying rates with daily egg weight.

### 4.4. Effect of Ginger on Reproductive Performance

Herve et al. [36] mentioned that the dose level of 100 and 150  µl ginger basic oil/kg BW, this basic oil caused a clear advancement in fertility ratio in comparison to the control outcomes. On the other side, Tchoffo et al. [45] assessed the outcomes of ginger roots extracted basic oil on some generative features of laying birds, 80 female Japanese quails were haphazardly allocated into 4 dietary categories (3 weeks of age). From the 3rd till 13th week, Japanese quails in group one, orally treated by clean H_2_O (100 µl/kg BW), all over the same time, orally gavaged 50, 100 and 150 μl of ginger roots basic oil per kg BW. No clear advances were noticed during the trial for body and individual ovary relative weights. While, the individual uteri mass clearly advanced in dose-associated pattern. In addition, fertility, hatchability, hatchability rate and chick’s weight clearly advanced in poultry treated by 100 and 150 µl/kg BW in relation to those of control data. Embryonic deaths dropped clearly with any level of the ginger rhizomes essential oil dose [45].

### 4.5. Effect of Ginger on Blood Parameters

Dieumou et al. [34] evaluated that the impact of garlic and ginger basic oils on some blood parameters of broiler chickens. Forty unsexed two-days old chicks of Arbor acres were allocated in four experimental groups. They were received via stomach tube either four doses; 0 (Control), 10 mg/kg/day, 20 mg/kg/day, and 40 mg/kg/day. The result showed no clear variations recorded in serum alanine transaminase (ALT) aspartate transaminases (AST) and serum creatinine features due to ginger treatments. 

Shanoon et al. [35] theorized that there were no obvious differentiations in serum transaminases (AST or ALT) and blood creatinine features, indicating that none of the examined three dosages of the oil that were administered to birds was harmful. Also, Herve et al. [36] confirmed that the serum contents of transaminases, malondialdehyde (MDA), triglycerides and total cholesterol were decreased in treated quails. The serum features in total protein, globulin and antioxidant enzymes were elevated in exposed birds considered to the control data. As well, Herve et al. [37] found that the serum features of total cholesterol, low density lipoprotein (LDL), and transaminases (AST or ALT) clearly dropped with 100 or 150 µl/kg BW of ginger roots extracted basic oil when compared to control data, as reported by also found that the oral application of 100 or 150 µl/kg BW of ginger roots extracted essential oil to laying Japanese quails greatly dropped cholesterols (serum or egg) levels without any harmful outcomes on feed uptake and BW profits.

Saleh et al. [46] monitored the outcomes of feed supplements of either thyme or ginger basic oils on hematological, biochemical and immunological features of broilers. A 105 one-day-old Ross 208 broilers in seven experimental groups. The control group received only the basic feed. The experimental feeds considering thyme oil at doses of 100 (T100), 200 (T200) and 300 (T300) mg/kg BW. Ginger oil at doses of 100 (G100), 200 (G200) and 300 (G300) mg/kg BW. The hematological outcomes cleared an obvious elevation in packed cell volume or hemoglobin in T200 group and in total leukocytes count especially heterophils percentage in both T200 and G100 groups. Total protein or globulin clearly dropped in G200 or G300 groups. While, group G100 indicated the better impact on lipid parameters.

Habibi et al. [38] demonstrated that the category was received 150 mg/kg BW ginger extracted basic oil, caused the total SOD function in hepatic tissue was elevated in relation to the control data. MDA values in hepatic tissue were diminished in the groups fed either ginger powder or basic oil in relation to that in the control data. Insignificant variations among treated groups considering, total SOD, glutathione peroxidase (GSH-PX) and catalase enzymes in red blood cells were recorded. All treated groups showed elevations in total antioxidant capacity (TAOC) or lowered MDA contents in serum in relation to the control data. Besides, Shanoon et al. [35] revealed that there were insignificant variations noticed in the serum transaminases (AST or ALT) and serum creatinine values, improving that neither of the these three levels of oil (10, 20 and 40 mg/kg BW /day) that were administered to birds was toxic.

Abd El-khalek and ELnaggar [40] found that addition of different medicinal plants either alone or in a mixture at any level significantly decreased plasma total cholesterol concentration and the activity of ALT compared to control. Also, Tchoffo et al. [45] confirmed that the values of serum total proteins, estradiol, follicle stimulating hormone and luteinizing hormone were greatly improved in a dose-associated pattern.

Al-Tahtawy et al. [47] examined the outcomes of *Zingiber officinale* Roscoe (ginger) extraction on hypercholesterolemic atherosclerosis condition due to expected antioxidant capacity. Data revealed a clear depression in the features of cholesterol, phospholipids, triglycerides and (VLDL or LDL) cholesterol levels in both serum and aortic tissue homogenate associated with high doses of ginger extraction. In addition, the antioxidant action of ginger extraction was estimated through its radical scavenging action (RSA), using rapid 1,1-diphenyl-2- picrylhydrazyl (DPPH) technique, indicated maximum antioxidative action associated with high doses of ginger extract.

An et al. [44] mentioned that ginger extraction did not modify the action of GSH-PX and TAOC, but only modified plasma SOD feature, decreased MDA value of the birds. In addition, ginger extract did not modify the values of serum total protein, albumin, globulin, but positively magnified lysozyme (LZM) action. On the same line, ginger extract clearly lessened prostaglandin E2 (PGE2) value. Moreover, Ghasemi and Taherpour [41] recognized that serum cholesterol values were diminished in the ginger essential oils (100 mg/kg) and mannan-oligosaccharide-supplemented diet.

Tekeli et al. [42] presented that blood cholesterol values, on the other side, blood glucose values was advanced by *Z. officinale*, at the same time blood triglyceride values was elevated by both *Z. officinale* and *S. aromaticum* dietary treatments.

The serum AST, ALT, total cholesterol and LDL-cholesterol were markedly decreased with 100 or 150 µl/kg BW of ginger roots extracted basic oil when considered with control data [37]. Oral application of 100–150 µl/kg BW of ginger roots extracted basic oil on laying Japanese quails, lowered serum cholesterol levels without any harmful factors on feed uptake and BW profits [36].

### 4.6. Effect of Ginger and Its Derivatives on Microbiological Aspects

Dieumou et al. [34] evaluate that *Escherichia coli*, like majority of Enterobacteriaceae populations in the ileo-cæcal contents quantitatively diminished in comparison with the control data in response to increased levels of ginger essential oils. The same measures were performed for the *Salmonella* and *Shigella* species. *Staphylococci spp* CFU (colony forming units) were nearly identical between ginger oil-treated groups, but were clearly diminished in comparison to the control data. Yeast and molds were present in the ileo-cæcal contents of all experimental groups. 

Sharma et al. [29] discovered that the ginger essential oil evidenced clear antimicrobial actions against selected bacterial (*Staphylococcus aureus*, *Escherichia coli* and *Pseudomonas aeruginosa*) and fungal (*Aspergillus niger* and *Candida albicans*) populations. Ginger basic oil primarily included large characters of monoterpenes as sesquiterpenes, and showed critical antimicrobial action against most pathogenic microorganisms.

Bhattarai et al. [31] discussed the antimicrobial action of 8% ginger extraction from fresh ginger and the residue ginger after essential oil extraction against selected bacterial populations (*Staphylococcus aureus*, *Klebsiella spp* and *Escherichia coli*) stated through the inhibition zone and compared with chloramphenicol data. Similarly, the antioxidant capacity was executed by DPPH radical scavenging technique in a wide variety of concentrations. Since, fresh ginger or the residue ginger after essential oil extraction displayed the maximum percentage inhibition as compared with ascorbic acid, but the essential oil did not have the antioxidant actions.

Norajit et al. [48] mentioned that ginger essential oil extracted by hydrodistillation possessed a maximum capacity towards three gram-positive bacterial strains, with a minimal concentration to suppress both *L. monocytogenes* and *B. cereus* of 6.25 mg/mL. Stoyanova et al. [32] evaluated the antimicrobial action of basic oils extracted from ginger herb by agar diffusion technique. The examined microorganisms were some gram-positive strains (*Bacillus subtilis*, *Bacillus pumilus*, *Staphylococcus aureus* and *Staphylococcus epidermidis*); some gram-negative strains (*Salmonella abony*, *Escherichia coli* and *Pseudomonas aeruginosa*); some yeast strains (*Candida albicans* and *Saccharomyces cerevisiae*) and some fungal strains (*Aspergillus niger*, *Botrytis cinerea*, *Penicillium spp*. and *Rhizopus nigricans*). The basic oil exhibited a little action over the examined gram-positive and gram-negative strains and possessed a potential fungicidal action on the examined fungal strains. Abd El-khalek and ELnaggar [40] suggested that the intestinal total anaerobic and aerobic bacterial counts as well as the counts of *coliforms* population minimized due to the addition of various medicinal herbs combined or singly whatever the concentration level comparable to control data.

Majolo et al. [49] quantified components and examined the antibacterial action of basic oils originated from roots of *Zingiber officinale* Roscoe or *Curcuma longa* L. The extraction techniques of basic oils were executed by recommended GC–MS option. The antibacterial action was executed with microdilution broth technique. The essential oil of ginger was clearly approved to be more effective than turmeric oil, both were considered of bacteriostatic impact (minimum inhibitory concentration 2500–5000 µg/mL) or bactericidal impact (minimum bactericidal concentration 5000–10000 µg/mL). So, ginger essential oil was considered as a substitute agent for governing of *Salmonella enterica* infection.

Tekeli et al. [42] concluded that dietary addition of *Z. officinale* improved beneficial bacteria population, like lactic acid bacterial population in the small intestine mainly jejunum. El-Baroty et al. [33] reported that ginger-extracted oil could show a significant suppressing action against some picked bacterial strains and some pathogenic fungi, with recorded minimum inhibitory concentration (MIC) levels extending from 20 to 120 µg/mL according to the bacterial strains. By increasing doses of oil, *Escherichia coli* or other Enterobacteria (*Salmonella* and *Shigella*) populations in the intestinal contents significantly dropped when compared with control data [35].

### 4.7. Effect of Ginger on Meat and Egg Quality

In previous literatures, the impacts of ginger or its derivatives on the meat quality and chemical composition of meat are rare. Ginger is an important source of the plant proteolytic enzyme. Naveena and Mendiratta [58] stated that the extracts of ginger have proteolytic activity, resulting in an increase in the solubility of collagen and proteolysis in this herb extract treated spent chicken muscle. On the other hand, Attia et al. [59] studied the effect of ginger on performance and cost of supplementation and they found that dietary supplementation of 0.5% ginger decreased meat lipids and plasma glucose in comparison with the control. Herawati and Marjuki [60] found that chickens given diets with ginger (0.5, 1.0, 1.5 and 2.0%) showed a significant decrease in the fat weight than birds given diet without ginger. Also, using ginger in broiler diets instead of chemical antibiotics slightly increased tenderness and pH of broiler meat, but decreased cooking loss and water holding capacity as compared to the control.

Ginger plays an important role in improving the egg quality, since, in Hyline Brown laying hens, dietary supplementation of ginger (100 g/ton for eight weeks) improved egg quality by increasing albumin height and Haugh unit of eggs than the control birds. Also, use of ginger in layer diets increased activity of T-SOD and decreased content of MDA and cholesterol in yolk than the control hens [61]. These results were consistent with the findings of Zhao et al. [57] who found that layer fed diets enriched with ginger powder decreased MDA concentration and increased T-SOD activity in egg yolk. Yang et al. [62] also stated that improved Haugh unit was accompanied by increased blood antioxidant enzymes in birds fed ginger root. This may be explained by the radical-scavenging effect of phenolic compounds and antioxidant components in ginger extract, which reduce lipid oxidation [63] and enhance the function of body organs as partly reflected by decreased the serum activities of ALT and AST, thus improving the production and synthesis of antioxidant enzymes [64]. On the same line, Gurbuz and Salih [65] found that powder of ginger root lowered yolk cholesterol content. The hypocholesterolemic effect of this plant was also recorded in broiler chickens [18]. Furthermore, the decrease in yolk content of cholesterol may be returned to the alterations of HDL-metabolism, which is involved in reverse the transport of cholesterol. Ginger extract may have used as a feed additive for increasing the production of low-cholesterol eggs, which would be preferred by consumers and customers because cholesterol is an indicator for cardiovascular diseases [66].

### 4.8. Effect of Ginger on Economic Efficiency 

Ginger powder also could be utilized in poultry to minimize cost-benefit ratio and improve economic feasibility. Oleforuh-Okoleh et al. [67] studied the effect of ground ginger on the growth performance, carcass quality and economics of production of broiler chickens. They found ground ginger in water-based infusion at 50 mL/liter of drinking water or ground ginger at 14 g/kg of the diet achieved the highest revenue and net return, and also gave the least cost-benefit ratio in comparison with the control [67]. Karangiya et al. [68] studied the effect of ginger, garlic and their combination on growth performance and economics in broilers. The income from selling of the birds was higher in 1% of ginger group than the control. During the whole experimental period, feed cost was higher in 1% of ginger group and 1+1 ginger and garlic group than the control. Return over feed cost was lower 1% of ginger group and 1+1 ginger and garlic group than the control. It could be concluded that ginger did not show any positive or negative impact on Return over feed cost. Also, this study was in accordance with Mohammed and Yusuf [69] who observed no differences in cost of feed per kg BWG for broiler chickens due to dietary supplementation of ginger.

## 5. Conclusions

Ginger and its derivatives could be mentioned harmless due to possessing no acute toxicological collateral outcomes as reviewed through the experimental time. According to many studies, data were mentioned that the experimental feed supplements minimized gizzard mass and abdominal fat, but without outcomes in broiler growth features and carcass profiles. It can be also stated that feed supplements with ginger exhibited a positive domination upon immune action and antioxidants of broilers. It could be due to ginger possessed intense antioxidant action, considering that microbial based dietary add-on enhanced regular antibodies formation. A variety of alternatives have been utilized as antibacterial agents in the poultry manufacturing. Use of ginger extract as a feed additive to increase the production of low-cholesterol eggs, which would be preferred by consumers and customers because cholesterol is an indicator for cardiovascular diseases. Many research results indicated that ginger and its derivatives showed similar effects to antibiotics in poultry. In addition, appropriate doses and the method of application for these alternatives to antibiotics are important in order for them to be more effective in poultry.

## Figures and Tables

**Table 1:** Beneficial effects of ginger and its derivatives in poultry nutrition.

Forms and Doses	Results	Author/s
Ginger oil (100 µl/kg body weight (BW))	Weight of eggs clearly improved in Japanese quails orally administered with ginger root extract	[36]
Ginger roots extracted basic oil (100 or 150 µl/kg BW)	The serum transaminases (alanine transaminase and aspartate transaminases), total cholesterol and LDL-cholesterol were markedly decreased	[36]
Ginger oil (40 mg ginger oil/kg/day)	By increasing doses of oil, *Escherichia coli* or other Enterobacteria (*Salmonella* and *Shigella*) populations in the intestinal contents significantly dropped when compared with control data	[35]
Mannan-oligosaccharide plus ginger essential oil (200 mg/kg diet) mixture	The birds nourished on this mixture showed an improved body weight gain (BWG) from the first to 42 days of age when compared with control data.	[41]
Basal diet + 120 mg, *Zingiber officinale* essential oil/kg	*Z. officinale* improved the BWG in comparison with control and other treated groups.	[42]
Ginger oil (0, 0.25%, 0.50% and 0.75%)	Egg mass clearly advanced in Japanese quails exposed to ginger roots extracted basic oil without concerning the level of the dose with special attention to the control data.	[43]
Oral application of 100–150 µl/kg BW of ginger roots oil.	Use of ginger root oil in laying Japanese quails recorded the highest outcomes on egg mass and lowered egg and serum cholesterol levels without any harmful factors on feed uptake and BW profits.	[36]
Ginger oil (100 and 150 µl/kg BW)	This oil caused a clear advancement in fertility ratio in comparison to the control outcomes.	[36]
Ginger oil (100 and 150 µl/kg BW)	Fertility, hatchability and chick’s weight were improved in birds treated by ginger oil. Embryonic deaths dropped clearly with any level of the ginger rhizomes essential oil dose. The values of serum total proteins, estradiol, follicle stimulating hormone and luteinizing hormone were greatly improved in a dose-associated pattern.	[45]
0.1% ginger extract	Ginger extract clearly advanced laying rates with daily egg weight	[44]
Ginger oil (100 and 150 l/kg BW)	Serum contents of malondialdehyde (MDA), triglycerides and total cholesterol serum features were decreased. Total protein, globulin and antioxidant enzymes were elevated.	[36]
Ginger oil at doses of 100, 200 and 300 mg/kg BW.	Ginger oil at dose of 100 mg/kg BW improved serum lipid profile.	[46]
150 mg/kg BW ginger extracted basic oil	Superoxide dismutase (SOD) in hepatic tissue was elevated by ginger compared to control data. MDA values in hepatic tissue were diminished in the groups feeding ginger powder oil.	[38]
Ginger extraction	Ginger extract clearly lessened prostaglandin E2 (PGE2) value.	[44]
Ginger essential oils (100 mg/kg)	Serum cholesterol values were diminished in the ginger essential oil and mannan-oligosaccharide supplemented diet.	[41]
*Z. officinale*	Blood glucose values were advanced by *Z. officinale*, at the same time blood triglyceride values was elevated by both *Z. officinale* and *S. aromaticum* dietary treatments.	[42]
Ginger essential oils.	*Escherichia coli* populations in the ileo-cæcal contents quantitatively diminished in comparison with the control data in response to increased levels of ginger essential oils.	[34]
Ginger essential oil	*Staphylococcus aureus, Bacillus subtilis, Escherichia coli* and *Pseudomonas aeruginosa* and fungal (*Aspergillus niger* and *Candida albicans*) populations were decreased with ginger oil supplementation.	[29]
8% ginger extraction from fresh ginger and the residue ginger after essential oil extraction.	*Staphylococcus aureus, Bacillus subtilis, Klebsiella spp* and *Escherichia coli* were decreased with ginger oil supplementation.	[31,49]
Ginger extracted oil	Ginger extracted oil could show a significant suppressing action against some picked bacterial strains.	[33]
Ginger oil for 30 days	Oral application of ginger oil for at least 30 days, critically improved glutathione reductase, glutathione and SOD values.	[26]
Ginger powder (0.15, 0.20 and 0.25%)	Antibody titre was higher in birds fed 0.25% ginger than other rations after seven days post injection. The counts of *Lactobacillus* in ileal content of birds fed 0.20 and 0.25% ginger were higher in comparison with the other treatments.	[50]
Ginger powder (receiving ginger capsules 3 g/day in 3 divided doses)	Supplementation of ginger powder significantly decreased the levels of total cholesterol, triglycerides, low density lipoprotein (LDL) and very-low density lipoprotein (VLDL).	[51]
Ginger essential oil (125 ppm)	In broiler chickens receiving ginger essential oil greater high density lipoprotein (HDL) and lower VLDL levels, whereas no significant difference was observed in LDL concentration. Ammonia concentration in ileum was the lowest in broiler fed with essential oil supplementation.	[52]
Ginger extract	Ginger extract enhances the serological response and had an antioxidant activity (both in vitro and *in vivo*) mainly attributed to pungent active principles like shogaols and gingerols.	[22]
Ginger rhizome (10 mg/kg)	In vitro, ginger extract showed antibacterial activity against Salmonella typhimurium, Pseudomonas aeruginosa, Candida albicans and Escherichia coli.	[53]
Basal diet plus 2 g/kg, 4 g/kg and 6 g/kg ginger powder	Ginger powder increased hemagglutination inhibition (HI) titre against Newcastle Disease virus. Also, ginger powder at 6 g/kg increased the leucocytes count and serum total protein, but decreased cholesterol and high-density lipoprotein (HDL) levels.	[54]
Aqueous extracts of ginger	Aqueous extract of ginger improved performance and plays an immune stimulant against Newcastle Disease.	[55]
0.1% and 0.2% ginger	Birds fed 0.1% and 0.2% ginger had better feed conversion ratio	[56]
Ginger powder (5, 10, 15, or 20 g/kg of diet) for 10 weeks.	Dietary supplementation of ginger powder at 15 or 20 g/kg enhanced performance and egg yolk and serum antioxidant status and improved dietary oxidation stability in a dose-dependent manner in laying hens.	[57]
Ginger powder (particle size of 300 µm) at the rate of 5 g/kg.	Ginger powder increased activities of SOD and glutathione peroxidase and reduced MDA content in the serum of birds.	[18]
